# Downregulation of GRK5 hampers the migration of breast cancer cells

**DOI:** 10.1038/s41598-019-51923-1

**Published:** 2019-10-29

**Authors:** Ann-Katrin Sommer, Mathias Falcenberg, Bojan Ljepoja, Thomas Fröhlich, Georg J. Arnold, Ernst Wagner, Andreas Roidl

**Affiliations:** 1 Pharmaceutical Biotechnology, Department of Pharmacy, Ludwig-Maximilians-Universität München, D-81377 Munich, Germany; 2 Department of Molecular Biology, Max-Planck-Institute of Biochemistry, D-82152 Planegg, Germany; 3 Laboratory of Functional Genome Analysis (LAFUGA), Gene Center, Ludwig-Maximilians-Universität München, D-81377 Munich, Germany

**Keywords:** Kinases, Breast cancer

## Abstract

Sunitinib is a multispecific kinase inhibitor and one of its targets is the kinase GRK5, which is regulating a multitude of G protein-coupled receptors (GPCRs). In this study we demonstrate that a decreased GRK5 expression induced by knock-down experiments or sunitinib treatment hampers the migration of cancer cell lines. A proteomic analysis revealed many pathways related to cell migration which were down regulated upon the GRK5 knock-down. Furthermore, we found in MDA-MB-231 breast cancer cells that the inhibition of migration is mediated by the GPCR gastrin releasing peptide receptor (GRPR) leading to a reduced expression of migration regulating downstream targets like CDC42 and ROCK1. An *in silico* Kaplan Meier analysis revealed that GRK5 and GRPR overexpression reduces the distant metastasis free survival in triple-negative breast cancer (TNBC) patients. Thus, we suggest a novel anti-migratory effect of impaired GRK5 expression which induces a negative feedback loop on GRPR signalling.

## Introduction

G protein-coupled receptors (GPCRs) are the largest group of cell surface receptors in humans and comprise of more than 800 members^1^. They are also known as seven-transmembrane receptors and are involved in many physiological processes like neurotransmission, metabolism, immune response, regulation of blood pressure and cardiac activity^2^. Therefore, a tight regulation of these signalling cascades is of great importance to avoid disease formation. One regulatory protein class are the AGC kinases to which the G protein-coupled receptor kinase (GRK) family belongs. These seven serine-threonine kinases can be categorized according to their structural properties into three subgroups: the visual subfamily (GRK1, GRK7), the GRK2 subfamily (GRK2, GRK3) and the GRK4 subfamily (GRK4, GRK5, and GRK6)^3,4^. In general, all GRKs regulate the GPCR signalling by phosphorylating agonist-activated GPCRs in the third cytoplasmic loop and/or the C-terminal tail. This leads to the recruitment of β-arrestins and subsequently to the uncoupling of the G proteins. This process is termed GPCR desensitization^5,6^. β-arrestins are also known to function as scaffold proteins that facilitate the internalization of GPCRs thus resulting in the recycling or lysosomal degradation of the receptor^7^.

Due to the fact that the seven GRKs regulate more than 800 GPCRs it is obvious that GRKs affect the signal transduction of complex signalling cascades which comprise of more than one GPCR. Therefore, aberrant expression of these kinases leads to malfunction of several GPCRs and consequently to diseases like diabetes, hypertension, cardiac dysfunction, Alzheimer’s disease and cancer^1,5^. Tumorigenesis was shown to be influenced mainly by GRK2 and GRK5^8–10^. These kinases often function as oncogenes in glioblastoma^11^, prostate^9,12^, pancreas^13^, non-small-cell lung^14^ and breast cancer^15^. According to the Human Protein Atlas, 50% of the analysed breast cancer patients possessed elevated GRK2 or GRK5 expression^16,17^, whereas the latter was shown to result in even worse prognosis concerning the 5-years survival rate. Nevertheless, the role of GRK5 in breast cancer has not been studied and the mechanism of action remains rather unclear. Breast cancer affects one of eight women during their lifetime and is estimated to be the second leading cause of cancer deaths in women in the United States in 2018^18^. Therefore, it is of great importance to identify novel predictive biomarkers and druggable targets associated with this disease.

Therefore, the aim of this study was to elucidate the function of GRK5 in breast cancer and investigate whether the targeting of GRK5 could have a beneficial effect on cancer treatment. Sunitinib is the most potent, approved small-molecule inhibitor (SMI) targeting GRK5^19,20^. Since 2006 sunitinib is approved by the FDA for the treatment of gastrointestinal stromal tumours, which are refractory or intolerant to imatinib treatment, and for advanced, metastatic renal cell carcinoma (mRCC)^21,22^. This multispecific kinase inhibitor was shown to mainly inhibit the receptor tyrosine kinases (RTKs) vascular endothelial growth factor receptor (VEGFR) and platelet derived growth factor receptor (PDGFR)^23^ thereby blocking the angiogenesis of tumours. Thus, we additionally investigated the effect of sunitinib on GRK5 and breast cancer cells *in vitro*.

## Results

### Analysis of different breast cancer cell lines reveals increased GRK5 expression in mesenchymal cells

**Figure 1:**
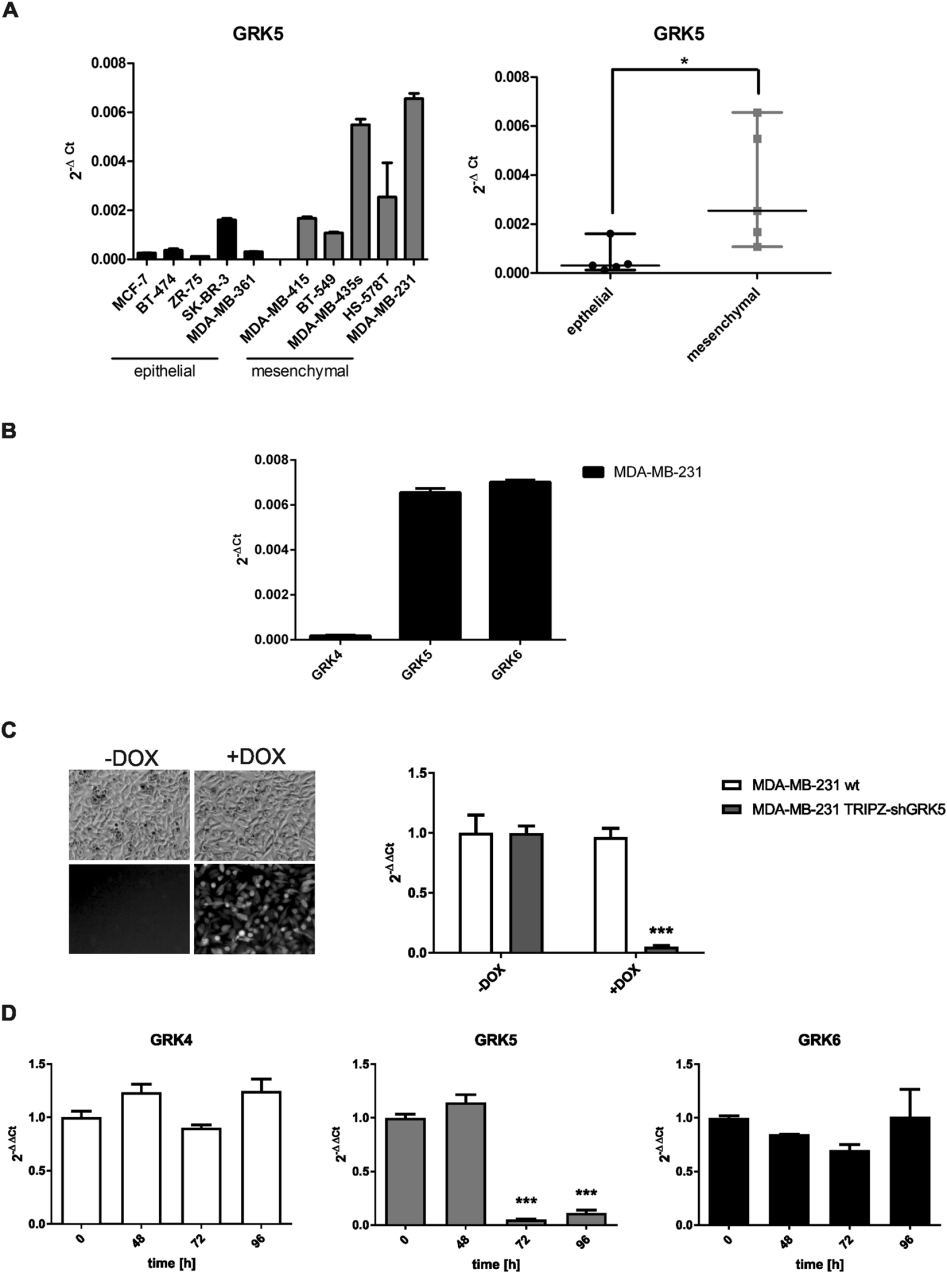
Expression analysis of GRK5 and characterization of MDA-MB-231 TRIPZ-shGRK5. (**A**) Expression analysis of GRK5 in different breast cancer cell lines utilizing qPCR. The values are depicted as mean + SD in the left panel and as median + range in the right panel. Student’s t-test was used for statistical evaluation. (**B**) Expression analysis of GRK4 family members in MDA-MB-231 wild-type cells. (**C**) Characterization of MDA-MB-231 TRIPZ-shGRK5. GRK5 expression analysis and fluorescence microscopy pictures upon induction with 5 µg/ml doxycycline for 72 h. (**D**) Expression analysis of GRK4 family members at the indicated time points upon induction with 5 µg/ml doxycycline. N = 3, *p < 0.05, **p < 0.01, ***p < 0.001.

To evaluate the expression level of GRK5 in breast cancer, a qPCR analysis was performed. The cell lines were grouped according to their morphology. Spindle-like cell lines were regarded as mesenchymal (MDA-MB-415, BT-549, MDA-MB-435s, HS-578T, MDA-MB-231) and cobblestone-like growing cells were considered as epithelial cells (MCF-7, BT-474, ZR-75, SKBR-3, MDA-MB-361). We observed that GRK5 is significantly higher expressed in mesenchymal-like breast cancer cells and that MDA-MB-231 cells, a metastatic, triple-negative breast cancer cell line, showed the highest GRK5 expression level of all analysed cell lines (Fig. 1A). A similar expression pattern was observed by western blot analysis in an additional panel of cancer cell lines (Supplementary Fig. S1). Therefore, MDA-MB-231 was chosen as model for further experiments.

The expression levels of the other GRK4-family members, GRK4 and GRK6, were analysed in MDA-MB-231 cells. Here, GRK6 is as highly expressed as GRK5, whereas GRK4 shows a very low expression level (Fig. 1B).

### Characterization of an inducible shGRK5 knockdown in MDA-MB-231 elucidates the impact of GRK5 on cell viability, apoptosis, migration and invasion in breast cancer

In order to further investigate the function of GRK5 in triple-negative breast cancer (TNBC), MDA-MB-231 cells stably expressing a doxycycline inducible shGRK5 were generated (termed MDA-MB-231 TRIPZ-shGRK5 in the following). As a gene coding for RFP is localized downstream of the doxycycline (DOX) inducible promotor, a fluorescence signal indicates shGRK5 expression upon DOX induction (Fig. 1C). Subsequently, we investigated the mRNA expression level of GRK5 in MDA-MB-231 TRIPZ-shGRK5 and MDA-MB-231 wild-type (wt) cells 72 h after inducing with DOX to determine the knockdown (KD) efficiency (Fig. 1C).

Moreover, possible compensatory effects of GRK4-family members were investigated in a time-dependent manner after DOX treatment and could be excluded as the mRNA expression levels of GRK4 and GRK6 remain similar after the GRK5 KD (Fig. 1D).

To elucidate the physiological impact of GRK5 KD in TNBC development and progression, cell viability and apoptosis induction were measured 90 h after DOX stimulation in MDA-MB-231 TRIPZ-shGRK5 cells. We observed that the GRK5 KD neither reduces the cell viability nor increases the apoptosis rate (Supplementary Fig. S2).

### Proteomic analysis and boyden chamber experiments reveal impact of GRK5 on cancer cell migration

**Figure 2:**
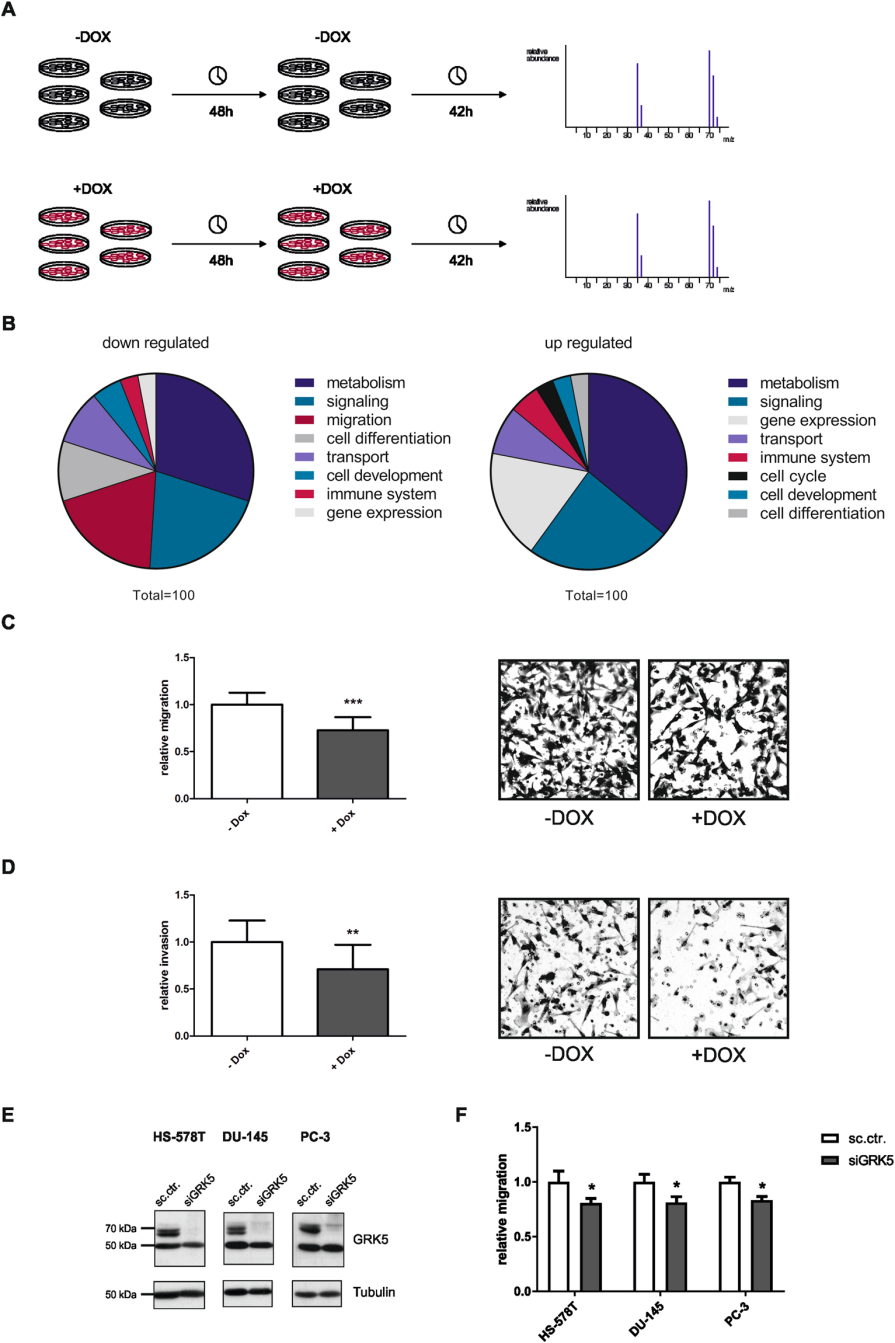
Proteomics, migration and invasion analysis of MDA-MB-231 TRIPZ-shGRK5. (**A**) Scheme of proteomics analysis. Cells were seeded in quintuples and stimulated with 5 µg/ml doxycycline or control every 48 h for 90 h. Subsequently a proteomic analysis utilizing LC-MS was performed. (**B**) Overview of up and down regulated pathways revealed by GSEA. Gene ontology biological process was utilized as dataset. (**C**) Migration analysis of MDA-MB-231 TRIPZ-shGRK5 90 h upon stimulation with doxycycline or control using a boyden chamber migration assay. Representative pictures are shown. (**D**) Invasion analysis utilizing matrigel coated boyden chambers 90 h after stimulation 5 µg/ml doxycycline or control. (**E**) Western blot analysis of HS-578T, DU-145 and PC-3 72 h upon siGRK5 KD. The presented blots were cropped (**F**) Migration analysis of HS-578T, DU-145 and PC-3 90 h upon transfection with sc.ctr. or siGRK5. Relative migration or invasion is shown as mean + SD normalized to not induced or sc.ctr. transfected samples (N = 3). A two-tailed Student’s t-test was performed. *p < 0.05, **p < 0.01, ***p < 0.001.

To investigate whether GRK5 influences breast cancer development and progression in general, a proteomics approach utilizing LC-MS was performed. Therefore, MDA-MB-231 TRIPZ-shGRK5 were seeded in quintuples, twice induced with doxycycline and harvested after 90 h (Fig. 2A). The subsequent proteomic analysis revealed 2220 proteins that were identified at least three times in induced and not-induced samples. The following gene set enrichment analysis (GSEA) utilizing Gene Ontology Biological Process (GO_BP) elucidated up and down regulated signalling pathways upon GRK5 KD. By clustering the resulting pathways in signalling cascades important for tumorigenesis it was observed that a decrease in GRK5 expression primarily leads to a down regulation of cell motility pathways (Fig. 2B).

To validate the effect of GRK5 on cancer cell migration, boyden chamber experiments with and without matrigel coating were performed. Thereby, we showed that GRK5 KD significantly hampers both migration and invasion in TNBC cells (Fig. 2C,D). Additionally, a KD by a pool of GRK5 siRNAs was performed in another breast cancer (HS-578T) and two prostate cancer cell lines (DU-145, PC-3) (Fig. 2E) and its effect on cancer cell migration was confirmed (Fig. 2F).

### GRK5 KD hampers chemotaxis of MDA-MB-231 cells towards bombesin

**Figure 3:**
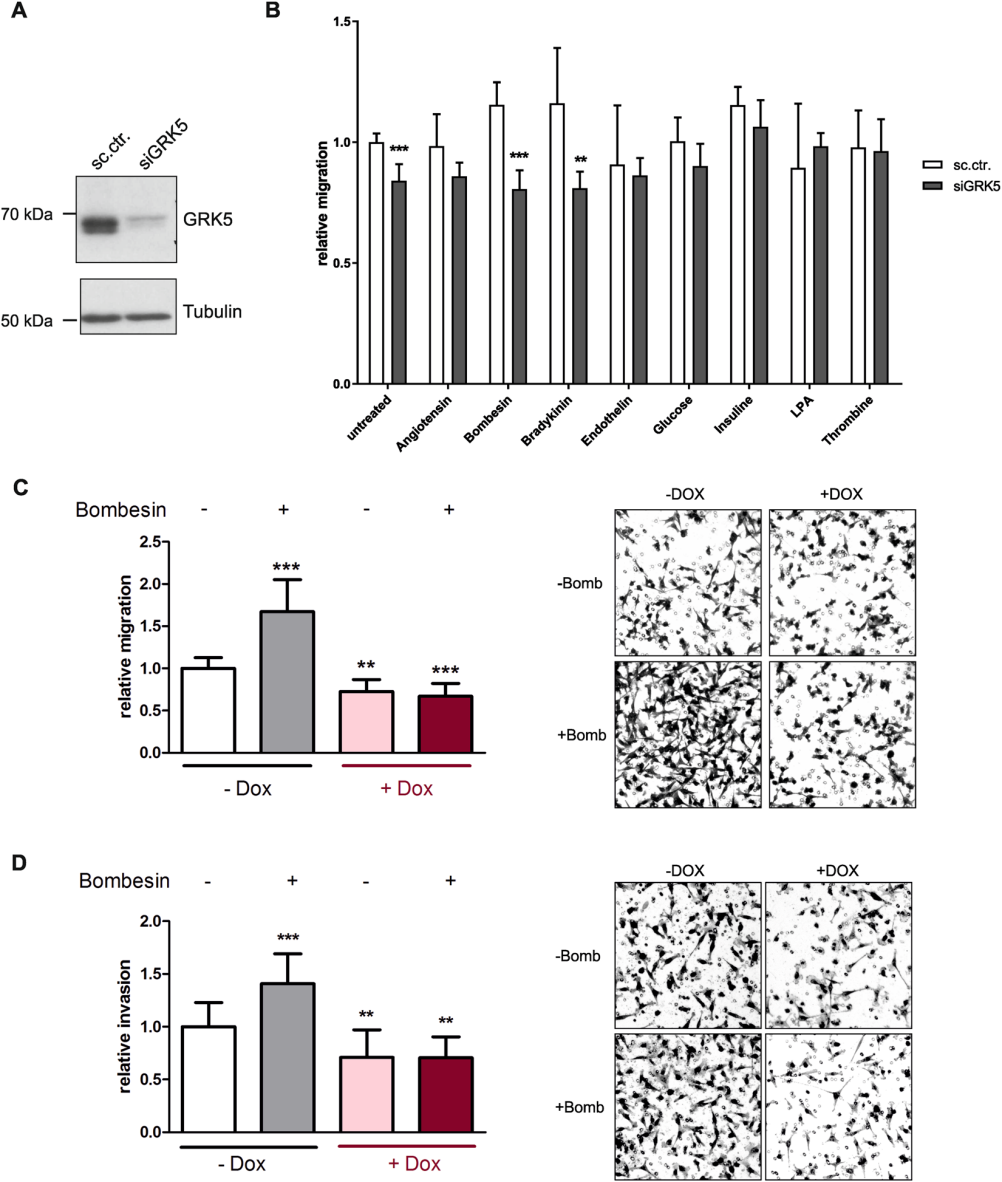
Evaluation of chemoattractants with regard to their migration stimulating capabilities in MDA-MB-231 cells and the blockage thereof by GRK5 KD. (**A**) Western blot analysis of MDA-MB-231 72 h upon siGRK5 KD. The presented blots were cropped. (**B**) Screening of different chemoattractants. The indicated ligands were added in the lower well at the following concentrations: Angiotensin 100 nM, Bombesin 200 nM, Bradykinin 5 µM, Endothelin 100 nM, Glucose 4.5 g/ml, Insulin 10 µg/ml, LPA 10 µM. Relative migration values are depicted as mean + SD and are normalized to untreated, sc.ctr. transfected cells (N = 3). (**C**) Analysis of migratory behaviour of MDA-MB-231 TRIPZ-shGRK5 cells 90 h upon induction with 5 µg/ml doxycycline or control with or without stimulation with 200 nM bombesin. Cells were stained with crystal violet and representative pictures are shown (N = 3). (**D**) Invasion analysis of MDA-MB-231 TRIPZ-shGRK5 90 h upon induction with 5 µg/ml doxycycline or control with or without stimulation with 200 nM bombesin. Cells were stained with crystal violet and representative pictures are shown. Values are depicted as mean + SD and are normalized to not induced, not stimulated samples (N = 3). One-way ANOVA with Dunnett’s Multiple Comparison Test and multiple t-tests utilizing two-stage linear step-up procedure of Benjamini, Krieger and Yekutieli was used for statistical analysis. *p < 0.05, **p < 0.01, ***p < 0.001.

To identify the underlying signalling pathways, different chemoattractants were analysed in a boyden chamber experiment with respect to their pro-migratory activity on MDA-MB-231 cells. Moreover, it was investigated whether this effect can be blocked or reduced by a siGRK5 KD. We demonstrate that only bombesin, bradykinin and insulin increased the migratory behaviour of MDA-MB-231 cells, which can be significantly decreased by a GRK5 KD in the case of bradykinin and bombesin (Fig. 3A,B). As bombesin shows the most significant change in migratory behaviour this ligand was analysed further.

The impact of bombesin on the migration of TNBC cells was also examined in MDA-MB-231 TRIPZ-shGRK5 cells to exclude transfection artefacts, as transfection agents could influence cancer cell migration. Here, the chemotactic effect of bombesin was also reduced by shGRK5 (Fig. 3C). Furthermore, the influence of GRK5 and bombesin on the invasiveness of MDA-MB-231 TRIPZ-shGRK5 cells was investigated. As depicted in Fig. 3D, a reduction of GRK5 expression leads to a decreased invasion of TNBC cells towards a bombesin gradient.

### Reduction in GRPR expression by GRK5 KD decreases chemotaxis of MDA-MB-231 towards bombesin

**Figure 4:**
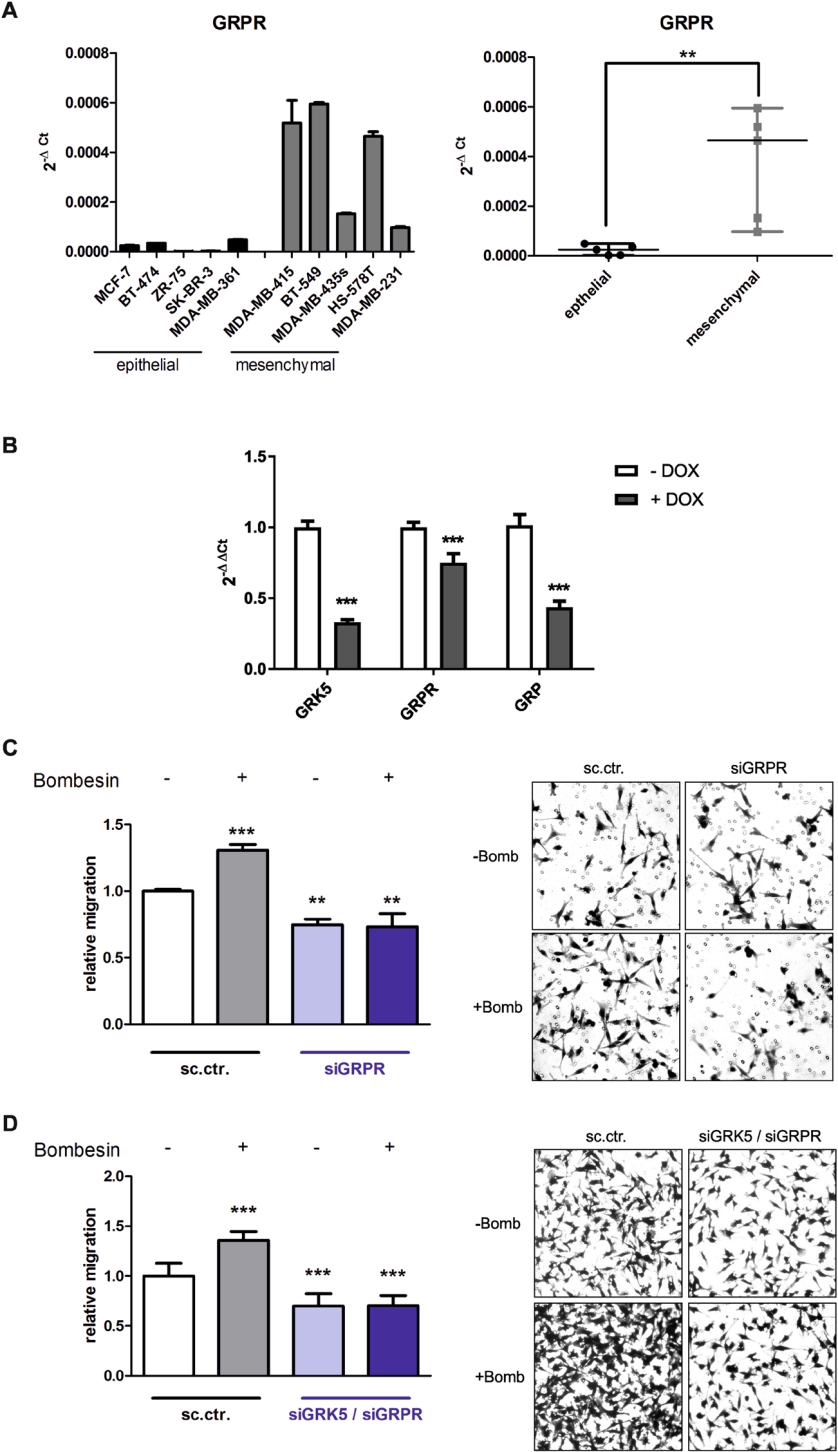
Correlation of GRK5 and GRPR and their impact on migration. (**A**) Expression analysis of GRPR in different breast cancer cell lines utilizing qPCR. The values are depicted as mean + SD in the left panel and as median + range in the right panel. Student’s t-test was used for statistical evaluation. (**B**) Expression analysis of GRK5, GRPR and GRP 90 h upon induction with 5 µg/ml doxycycline utilizing qPCR. Values are depicted as mean + SD. Two-tailed Student’s t-test was utilized for statistical evaluation. (**C**) Migration analysis of MDA-MB-231 cells 90 h upon transfection with sc.ctr. or siGRPR with and without stimulation with 200 nM bombesin. Representative pictures of the crystal violet staining are shown at the right panel. Relative migration values are depicted as mean + SD and normalized to sc.ctr. transfected, not stimulated samples. (N = 3). (**D**) Migration analysis of MDA-MB-231 cells 90 h after transfection with sc. ctr. or siGRK5 and siGRPR, with or without bombesin stimulation using a boyden chamber assay. Representative pictures of the crystal violet staining are shown. The relative migration values are normalized to sc.ctr. transfected, not stimulated samples and depicted as mean + SD (N = 3). For statistical evaluation one-way ANOVA with Dunnett’s Multiple Comparison Test was used. *p < 0.05, **p < 0.01, ***p < 0.001.

The gastrin releasing peptide receptor (GRPR) is the cellular receptor of bombesin and therefore, its expression levels were analysed in different breast cancer cells to confirm its impact on migration. Figure 4A shows that GRPR is significantly higher expressed in mesenchymal-like breast cancer cells. Subsequently, the influence of the GRK5 KD on the GRPR signalling pathway was investigated. Here, we observed that the autocrine activation loop of the GRPR signalling is hampered upon GRK5 KD since the mRNA level of GRPR as well as that of its natural ligand GRP was reduced (Fig. 4B). A GRPR KD on the other hand had no impact on the GRK5 expression (Supplementary Fig. S3). Next, the impact of GRPR on cell migration of TNBC was examined. We detected that GRPR KD reduces the migratory behaviour of MDA-MB-231 to a similar extent as a GRK5 KD (Fig. 4C). In order to determine whether GRPR is the only GPCR that is regulated by GRK5 and thus is responsible for the reduced chemotaxis towards bombesin, cells were transfected with both siGRK5 and siGRPR. As depicted in Fig. 4D this double-KD is not further reducing the migration of MDA-MB-231 cells indicating that GRPR is the main player in the chemotactic process which is regulated by GRK5.

By comparing the measured migration data, we observed a constant reduction in the migratory behaviour of 25–30% upon GRK5 KD, GRPR KD and the double KD of GRK5 and GRPR emphasizing the key role of GRPR in GRK5 mediated migration.

### Sunitinib treatment hampers cell migration by reducing the expression of GRK5 and GRPR

**Figure 5:**
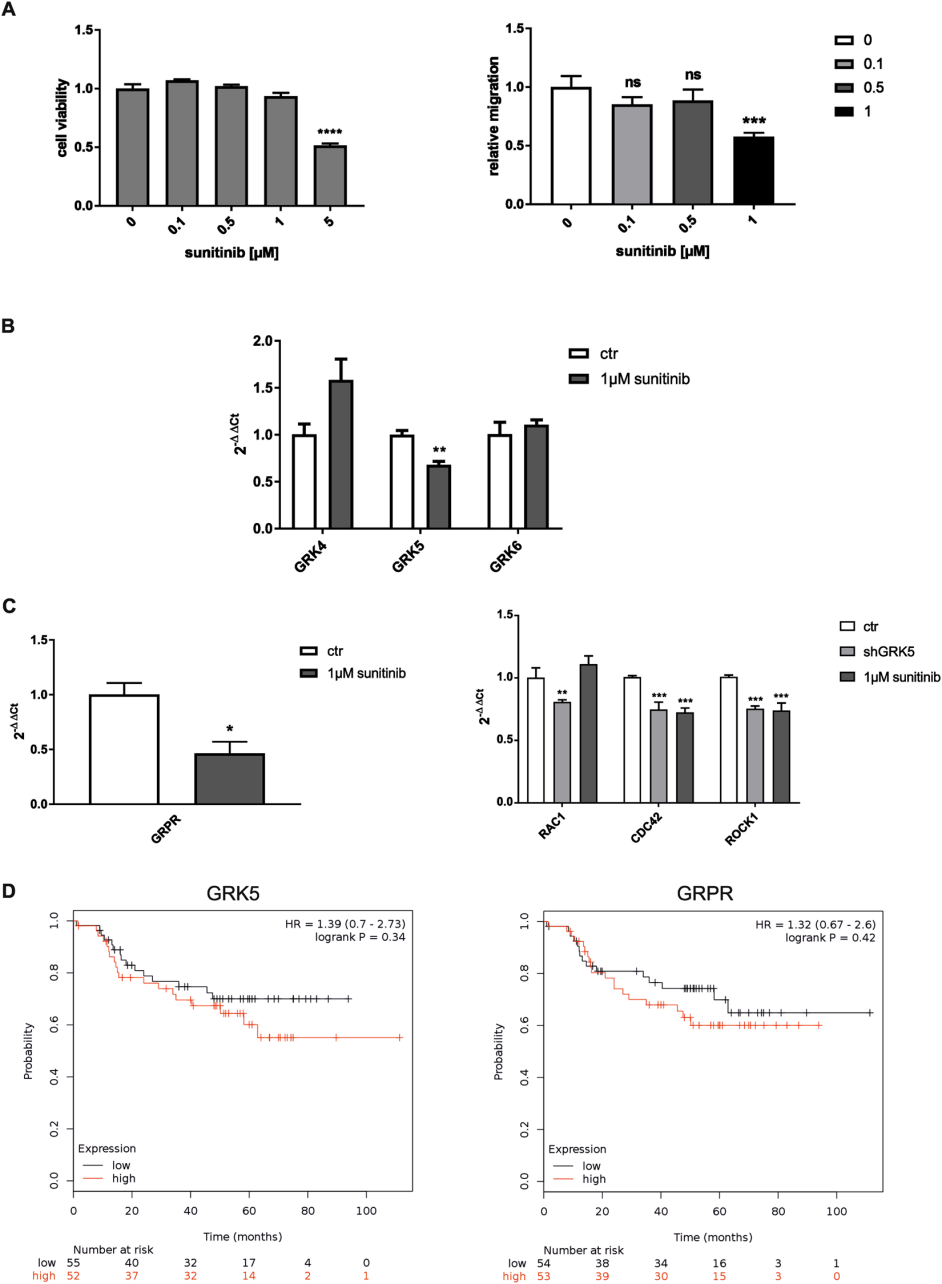
Analysis of clinical impact. (**A**) MDA-MB-231 cells were treated for 90 h with the indicated concentrations of sunitinib. Left panel: Cell viability measurement by CellTiter-Glo luminescent cell viability assay. Right panel: Migration analysis by boyden chamber. Values are presented as mean + SD and are normalized to untreated cells. For statistical evaluation one-way ANOVA with Dunnett’s Multiple Comparison Test was used (N = 3). (**B**) Gene expression analysis of GRK4-family members. Values are presented as mean + SD and are normalized to untreated cells. For statistical evaluation multiple t-tests utilizing two-stage linear step-up procedure of Benjamini, Krieger and Yekutieli, were used (N = 3). (**C**) Gene expression of GRPR and GRPR down-stream signaling components. Values are presented as mean + SD and are normalized to untreated cells. For statistical evaluation student’s t-test (left panel) and multiple t-tests (right panel) utilizing two-stage linear step-up procedure of Benjamini, Krieger and Yekutieli, were used. (**D**) Kaplan Meier analysis^24^ of GRK5 and GRPR overexpression. The influence on distant metastasis free survival (DMFS) in breast cancer patients with basal like tumours is depicted. *p < 0.05, **p < 0.01, ***p < 0.001.

**Figure 6:**
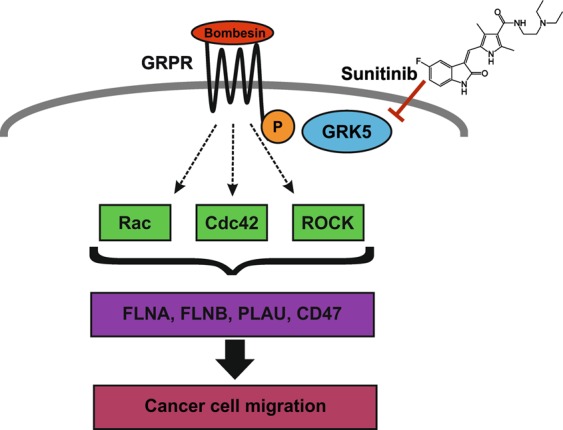
Supposed mechanism. GRK5 phosphorylates the GRPR upon bombesin binding. This leads to the activation of GRPR down-stream signalling and subsequently to the transcription of proteins like FLNA, PLAU and CD47. The final consequence is the maintenance of cell polarity and tension enabling cell migration which could be therapeutically exploited by inhibiting this pathway with sunitinib treatment.

Sunitinib is the most potent, approved GRK5 inhibitor^19,20^. To evaluate the clinical relevance of our findings MDA-MB-231 cells were treated with this SMI and its effect on cell viability, migration, GRK5 and GRPR expression as well as GRPR signalling was investigated. Figure 5A shows that 5 µM sunitinib treatment reduces the cell viability of TNBC cells. Therefore, only lower, non-toxic concentrations were utilized for the cell migration analysis. Here, we demonstrated that 1 µM sunitinib treatment significantly reduces the migration of breast cancer cells. A similar effect was detected for the GRK5 KD. The impact of sunitinib treatment on the expression of GRK4-family members was investigated. Sunitinib significantly reduces the expression only of GRK5 - no significant expression changes were observed for GRK4 and GRK6 (Fig. 5B). Additionally, sunitinib significantly decreases the expression of GRPR and down-stream targets like CDC42 and ROCK1 (Fig. 5C). To investigate whether the GRK5 inhibition, which leads to impaired GRPR signalling and eventually reduced migration, has an impact on patient survival, we analysed patient data *in silico*. The Kaplan-Meier Plotter^24^ revealed that GRK5 and GRPR overexpression shows a tendency to reduce the distant metastasis free survival (DMFS) in TNBC patients (Fig. 5D). Therefore, we hypothesize that sunitinib treatment of TNBC prolongs DMFS and thus could improve the patient outcome (Fig. 6).

## Discussion

Previous studies have shown that GRK5 affects the migration of prostate cancer via moesin^12^ and of non-small cell lung cancer cells via vinculin^14^. However, the function of GRK5 in breast cancer and the involved GPCRs remain unclear. In this study, a breast cancer cell line screen revealed that GRK5 is mainly expressed in TNBC cell lines, which possess a mesenchymal like phenotype and are able to migrate. Accordingly, the GRK5 KD by a pool of siRNAs and the inducible shRNA in MDA-MB-231 cells reduced cell migration and invasion as well as the expression of proteins involved in cell migration pathways. In order to uncover the responsible GPCRs different ligands were used as chemoattractants. Here, bombesin stimulation, amongst others, resulted in significantly increased cell migration which can be blocked by the GRK5 KD, mediated by both, the siRNA and shRNA approach. This finding led to the assumption that GRK5 interferes with the GRPR signalling pathway, as bombesin, an artificial ligand, binds and activates the GRPR^25–27^. GRPR is an important receptor in breast cancer and is overexpressed in up to 96% of breast cancer patients^28^. An elevated GRPR expression in breast cancer tissue worsens the prognosis^16,17^. Our expression analysis in different breast cancer cell lines revealed that GRPR is significantly higher expressed in mesenchymal like breast cancer cell lines and correlates with the GRK5 expression. The subsequent qPCR analysis of GRPR and its natural ligand GRP upon GRK5 KD displayed decreased GRPR and GRP expression. Vice versa the KD of GRPR had no influence on the GRK5 expression. Thus, the GRK5 KD hampers the autocrine signalling mechanism by inhibiting the expression of both the endogenous ligand as well as that of the corresponding receptor. GRPR is the sole receptor involved in the chemotaxis of cancer cells towards the ligands GRP/bombesin^29,30^. The decrease in its expression by GRK5 KD therefore directly impacts the migration of cancer cells. It was previously shown that the increase in GRPR signalling leads to enhanced cancer cell migration and invasion in various cancer types^31,32^. However, there are many more GPCRs involved in migration like the bradykinin receptor^33^, angiotensin receptor^34^, endothelin receptor^35^ and thrombin receptor^36^.Our ligand screen however revealed, that only bradykinin and bombesin stimulated migration was hampered by the GRK5 KD and are thus the crucial pathways influenced by GRK5. Moreover, we performed a KD with siRNAs against GRPR and a double KD with siRNAs against GRK5 and GRPR, and investigated the effect on migration. It was shown that all analysed KD experiments with siGRK5, shGRK5, siGRPR and the double KD of GRK5/GRPR, had the same impact on cell migration. We therefore conclude, that the GRPR signalling pathway is an important migratory mechanism which is regulated by GRK5. The knock-down of GRK5 activates a negative feedback loop by attenuating the expression of GRP and GRPR, finally leading to reduced migration in our *in vitro* system.

To evaluate the clinical significance of our findings, TNBC cells were treated with sunitinib, the most potent, FDA approved GRK5 inhibitor^19,20^. We observed that sunitinib hampers the migration of MDA-MB-231 cells at non-toxic doses. Previously, it was already shown that sunitinib hampers cell migration in different cancer subtypes but only at toxic doses^37,38^. Thus, these studies hardly allow a clear discrimination between cytotoxicity and migration. Furthermore, we performed an expression analysis of all GRK4-family members, GRPR and GRPR down-stream signalling components to elucidate whether the effect of sunitinib on cancer cell migration is based on the GRK5-GRPR signalling cascade. As sunitinib is a multispecific kinase inhibitor this SMI inhibits besides GRK5 e.g. VEGFR and PDGFR^19,20,38,39^. Our results show, that sunitinib treatment not only inhibits GRK5 but also significantly reduces its expression whereas GRK4 and GRK6 expression remains stable. Additionally, we observed that sunitinib treatment reduced the expression of GRPR and down-stream signalling components. As GRPR is no reported target gene of sunitinib, it is likely that sunitinib decreases the expression of GRK5 thus indirectly leading to the downregulation of GRPR and its downstream targets RAC1, CDC42 and ROCK1. The latter three proteins belong to the Rho GTPase family and are crucial players in cell migration^40,41^. Previous studies have shown that increased CDC42 and ROCK1 expression directly correlates with elevated actomyosin contractility, actin turnover and actin polymerization and eventually facilitate the migration of cancer cells^42^. Thus, sunitinib treatment of TNBC cells might reduce their ability to migrate by down regulating GRK5 resulting in the decreased expression of GRP, GRPR, CDC42 and ROCK1. Moreover, this finding might mechanistically explain the prolonged survival of mRCC patients upon sunitinib treatment^43^. Here, this therapy not only reduces the metastatic burden but also avoids the development of new metastases and thus leads to an improved patient outcome.

Taken together, this study shows that GRK5 KD hampers the chemotaxis of MDA-MB-231 cells towards bombesin by down regulating the GRPR. Furthermore, we observed that treatment with the multispecific kinase inhibitor sunitinib decreases the cancer cell migration by reducing the GRK5 expression levels resulting in attenuated GRPR signalling, depicting a novel mechanism of action of a well-known drug. We therefore encourage further studies on this mechanism and speculate, that the implementation of sunitinib in TNBC treatment regimen could be a promising option to reduce the formation of metastases which is still one of the major obstacles in the treatment of TNBC.

## Materials and Methods

### Reagents

Doxycycline hyclate was purchased from Sigma-Aldrich (St. Louis, Missouri, USA) (cat.nr. D9891). Bombesin acetate salt hydrate (cat.nr. B4272), Bradykinin acetate salt (cat.nr. B3259), human angiotensin II (cat.nr. A9525), endothelin I (cat.nr. E7764), lysophosphatidic acid sodium salt (cat.nr. L7260), human thrombin (cat.nr. T4393), glucose (cat.nr. D7021) and human insulin (cat.nr. I3536) were purchased from Sigma-Aldrich. Sunitinib malate was purchased from Sigma-Aldrich (cat.nr. PZ0012). Lipofectamine 3000 was purchased from ThermoFisher Scientific (Waltham, Massachusetts, USA) (cat.nr. L3000008).

### cDNA of different breast cancer cell lines

The cDNA of the different breast cancer cell lines was a kind gift of Axel Ullrich’s lab.

### Cell culture

MDA-MB-231 cells were obtained from DSMZ (Braunschweig, Germany) MDA-MB-231 TRIPZ-shGRK5 were generated in our lab and both were cultured in DMEM high glucose supplemented with 10% fetal calf serum (FCS, Gibco) at 37 °C and 5% CO_2_. HS-578T, DU-145 and PC-3 were obtained from ATCC (Manassas, Virginia, USA) and cultured according to manufacturer’s instructions. All cells were authenticated according to ANSI/ATCC standard ASN-0002 and routinely tested and confirmed as mycoplasm free.

### Generation of stable MDA-MB-231 TRIPZ-shGRK5

MDA-MB-231 cells were transduced with the doxycycline-inducible TRIPZ-shGRK5 [Clone-ID: V3THS_312367; Sequence: TCGTGAGCAGCATCTTGCA (Dharmacon)] construct utilizing a 2^nd^ generation lentiviral system generated with the plasmids pCMV-dR8.2 dvpr (Addgene plasmid # 8455) and pCMV-VSV-G (Addgene plasmid # 8454), which were a gift from Bob Weinberg^44^. After transduction, a 48 h selection with 5 µg/ml puromycin was performed.

Stimulation of the cells with doxycycline was performed in a concentration of 5 µg/ml in DMEM high glucose + 10% FCS for 90 h for mRNA, protein, migration and invasion analysis. Medium was replaced with fresh, doxycycline containing medium every 48 h to compensate for doxycycline degradation.

### siRNA transfection

For siRNA transfection 300 000 cells/well were seeded in a 6-well plate and transfected at the same time with 5 µl Lipofectamine 3000 and 12.5 pmol siRNA per well.

siGRK5: SMARTpool: ON-TARGETplus Human GRK5 siRNA (L-004626-02, Dharmacon, Lafayette, Colorado, USA)

scramble control: ON-TARGETplus Non-targeting Pool (D-001810-10, Dharmacon)

siGRPR: Silencer Select siGRPR s6230 (4392420, Thermo Fisher)

### RNA-lysis and purification

90 h prior to RNA lysis cells were seeded at a confluence of 50% and either transfected with siRNA or stimulated with doxycycline. Subsequently cells were harvested using RNeasy Mini Kit (Qiagen, Venlo, Netherlands) following the manufacturer’s protocol.

### cDNA synthesis

Upon RNA purification 1000 ng RNA were taken to synthesize cDNA according to manufacturer’s protocol using qScript cDNA Synthesis Kit (Quantabio, Beverly. Massachusetts, USA).

### Quantitative polymerase chain reaction (qPCR)

To analyse the mRNA expression a qPCR was performed using the LightCycler 480 (Roche, Basel, Switzerland), the Universal Probe Library (UPL, Roche) and LighCycler 480 Probes Master (Roche). The examined mixture contained 10 µl Probes Master, 4.4 µl nuclease free water, 0.2 µl left primer (20 µM), 0.2 µl right primer (20 µM), 0.2 µl probe and 5 µl cDNA per well. The utilized primer probe pairs are listed in the table below.

**Table:** 

	left primer	right primer	probe
GRK5	aagtccatctgcaagatgctg	ggggtgtctcttgacctctg	# 26
GRPR	cccgtggaagggaatataca	gcggtacaggtagatgacatga	# 36
GRP	cagccacctcaacccaag	tggagcagagagtctaccaactt	# 61
ROCK1	gatcccaaatcggaagtgaa	caaatcatataccaaagcatccaa	# 42
CDC42	tggagtgttctgcacttacaca	ggctcttcttcggttctgg	# 37
RAC1	ctgatgcaggccatcaagt	caggaaatgcattggttgtg	# 77
GAPDH	tccactggcgtcttcacc	ggcagagatgatgaccctttt	# 45

For quantification the 2^−ΔCt^ or the 2^−ΔΔCt^ method was applied and GAPDH was used as an internal standard.

### Protein lysis and western blot

Cells were lyzed at 80% confluence or 72 h after siRNA transfection with RIPA buffer containing 1% Triton X. 30 μg protein were separated using a SDS-PAGE and subsequently transferred to a nitrocellulose membrane. After one hour blocking with TRIS-buffered saline with Tween20 (TBST) containing 3% nonfat dry milk, the blots were incubated overnight at 4 °C with GRK5-antibody (Millipore, Burlington, Massachusetts, USA, cat.nr. 05-466) solution in TBST containing 3% nonfat dry milk, followed by several washing steps. Afterwards, membranes were incubated for one hour in horseradish peroxidase conjugated anti-mouse (goat anti-mouse-hrp, Sigma Aldrich) secondary antibody at room temperature. After additional washing steps, detection was performed using enhanced chemiluminscence (ECL, GE Healthcare, Chicago, Illinois, USA) on X-ray films (Amersham Hyperfilm ECL, GE Healthcare). The α-Tubulin antibody (Sigma Aldrich, cat.nr. T9026) was used as loading control.

### Evaluation of cell viability and apoptosis induction

To determine the impact of doxycycline and the GRK5 KD on cell viability the CellTiter-Glo luminescent cell viability assay (Promega, Madison, Wisconsin, USA) was utilized. To assess the apoptosis induction upon doxycycline treatment and GRK5 KD the Caspase-Glo 3/7 Assay (Promega) was used. 90 h prior to both measurements 3 000 cells/well of MDA-MB-231 TRIPZ-shGRK5 cells were seeded in 96-well plates and treated with the indicated concentrations of doxycycline (N = 3).

### Proteomics sample preparation

For proteomics analysis 300 000 cells/well were seeded (N = 5) in a 6-well plate and stimulated every 48 h with 5 µg/ml doxycycline for 90 h. Subsequently, cells were washed three times with cold PBS and lysed with a buffer containing 8 M urea and 400 mM ammonium bicarbonate. Ultrasound was used to support cell lysis and finally the protein samples were purified using QIA-shredder devices (Qiagen). For reduction, 30 µg of total protein was incubated for 30 min at a final concentration of 5 mM dithioerythritol (DTE). Cleaved bisulfide bonds were blocked using iodoacetamide (final concentration 15 mM) for 30 min in the dark. After dilution with water to a concentration of 1 M urea, proteins were first digested for 4 h with 300 ng LysC (FUJIFILM Wako Pure Chemicals, Osaka, Japan) and subsequently digested over night with 600 ng porcine trypsin (Promega, Madison, WI, USA) at 37 °C. Peptides were separated and identified on an Ultimate 3000 (Thermo Scientific, Waltham, MA, USA) nano-chromatography system coupled to a QExavtive HF-X instrument (Thermo Scientific). 2.5 µg of peptides were dissolved in 15 µl solvent A (0.1% formic acid in water) and transferred to a capillary trap column (PepMap 100 C18, 100 µm × 2 cm, 5 µM particles, Thermo Scientific). Separation was performed at 250 nL/min (Column: PepMap RSLC C18, 75 µm × 50 cm, 2 µm particles, Thermo Scientific) with a 160 min gradient from 5% solvent A to 25% solvent B (0.1% formic acid in acetonitrile) and a subsequent 10 min gradient from 25% to 40% solvent B. MS spectra were acquired using a top 15 data dependent CID method. Precursor spectra were acquired at a resolution 60,000 (mass-range: 350–1600) and MS/MS spectra at a resolution of 15,000.

### Migration and invasion analysis

For migration and invasion analysis cells were transfected with siRNA or stimulated with 5 µg/ml doxycycline 72 h prior to the experiment. Subsequently, 750 µl DMEM high glucose supplemented with 0.5% FCS and the indicated ligand was added to the lower well of the boyden chamber system Corning BioCoat Matrigel Invasion Chamber (Corning, Corning, New York, USA) in the case of invasion analysis and Falcon Cell Culture Inserts ([8 µm pores] Coring) in the case of migration analysis. The inserts (N = 3) were filled with 500 µl of a cell suspension containing 50 000 cells in DMEM high glucose supplemented with 0.5% FCS. 18 h later the cell suspension was removed and the inserts were put into 500 µl crystal violet solution containing 20% methanol for at least 10 min. Finally, the inserts were washed three times with demineralized water and kept overnight at room temperature. To determine the amount of migrated/invaded cells five microscopic pictures (magnification 10x) of each insert were made (top, bottom, left, right, center) and afterwards analysed using the ImageJ software.

### Sunitinib treatment

For cell viability measurement 3 000 MDA-MB-231 cells/well were seeded in triplicates in a 96-well plate and treated with the indicated concentrations for 90 h. Afterwards the CellTiter-Glo luminescent cell viability assay (Promega) was utilized to measure cytotoxicity. To analyse the impact of sunitinib on migration 300 000 cells/well were seeded in a 6-well plate and pre-treated with sunitinib at the indicated concentrations for 72 h. Subsequently, sunitinib was added to the starvation medium (DMEM + 0.5% FCS) for the following boyden chamber experiment. Here, 50 000 cells/well were seeded in triplicates to the insert. For gene expression analysis 300 000 MDA-MB-231 cells/well were seeded in 6-well plates and treated with 1 µM sunitinib for 90 h.

### Bioinformatics

Statistical significance was calculated utilizing a two-tailed Student’s t-test for the comparison of two samples and one-way ANOVA with Dunnett’s Multiple Comparison Test or multiple t-tests utilizing two-stage linear step-up procedure of Benjamini, Krieger and Yekutieli,for the comparison of several samples with the control. *p < 0.05, **p < 0.01, ***p < 0.001.

To process the mass spectrometry (MS) data MaxQuant 1.5.1.0 was applied^45^ and the Perseus module was used^46^. For label free quantification (LFQ) values of the identified proteins were taken and proteins that were only identified by site or potential contaminants were excluded.

The doxycycline induced (+DOX) and the not induced (−DOX) samples were grouped and at least three valid values in each group were necessary to enter further analysis. The missing values were replaced from normal distribution using the imputation feature of Perseus (width, 0.3; down-shift, 1.8). Subsequently, a gene set enrichment analysis (GSEA) was performed using gsea2-2.2.3 from the Broad Institute (Cambridge, MA, USA)^47,48^. As underlying gene set database the gene ontology biological process (GO_BP) was used^49^. The resulting, differentially expressed pathways were grouped in the following categories: metabolism, signalling, migration, cell differentiation, transport, cell development, immune system, gene expression and cell cycle to allow a comparison of up and down regulated signalling cascades.

In order to elucidate the clinical significance of GRK5 and GRPR in breast cancer patients the Kaplan-Meier-Plotter was utilized^24^ (released 2018/05/01). This web service allows the evaluation of more than 20,000 genes in about 1,800 breast cancer patients. MDA-MB-231 cells represent an example for a triple-negative breast cancer with a basal subtype according to its gene expression profile. Thus, patients with basal like tumours were analysed and the filters were set accordingly. Distant metastasis free survival (DMFS) was chosen as endpoint as cancer cell migration and invasion were of special interest in this study.

## Supplementary information

Supplementary figures S1, S2, S3

## Data Availability

Data for GSEA are available at 10.6084/m9.figshare.7415633.
