# Supplementary material for: Downregulation of GRK5 hampers the migration of breast cancer cells
Source: Sci Rep. 2019 Oct 29;9:15548. doi: 10.1038/s41598-019-51923-1 (PMC6820534; doi:10.1038/s41598-019-51923-1)
Supplement: Supplementary file 1 — Supplementary figures S1, S2, S3 [file 41598_2019_51923_MOESM1_ESM.pdf]

Downregulation of GRK5 hampers the migration of breast cancer cells

Supplementary information

Ann-Katrin Sommer [1], Mathias Falcenberg [2], Bojan Ljepoja [1], Thomas Fröhlich [3], Georg J. Arnold [3], Ernst Wagner [1] and Andreas Roidl [1,*]

[1] Pharmaceutical Biotechnology, Department of Pharmacy, Ludwig-Maximilians-Universität München, D-81377 Munich, Germany;

[2] Department of Molecular Biology, Max-Planck-Institute of Biochemistry, D-82152 Planegg, Germany;

[3] Laboratory of Functional Genome Analysis (LAFUGA), Gene Center, Ludwig-Maximilians-Universität München, D-81377 Munich, Germany

* Correspondence: andreas.roidl@cup.uni-muenchen.de; Tel.: +49-89-2180-77456

Supplementary figures

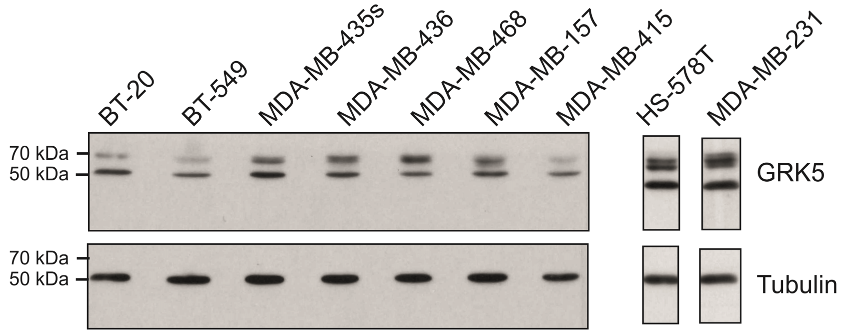

Supplementary figure S1. Western blot analysis of GRK5 protein expression in different breast cancer cell lines. Tubulin was used as loading control.

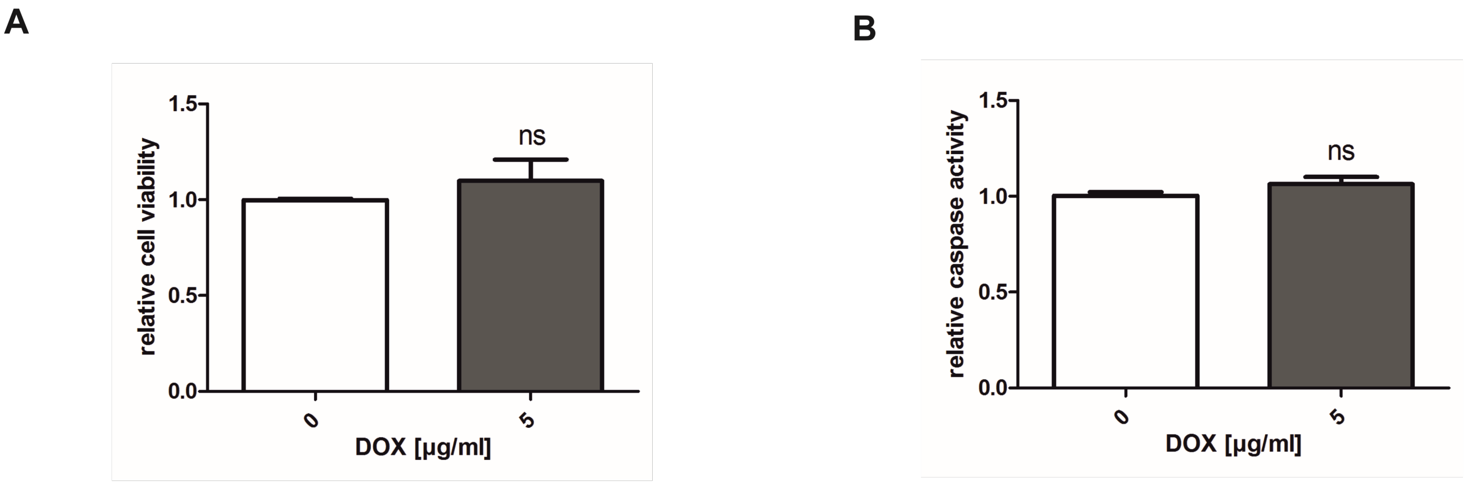

Supplementary figure S2. Cell viability and apoptosis measurement in MDA-MB-231 TRIPZ-shGRK5

(a) CellTiter-Glo Luminescent Cell Viability Assay 90h after treatment with 5 µg/ml DOX.

(b) Caspase-Glo 3/7 Assay 90h after induction with 5 µg/ml DOX.

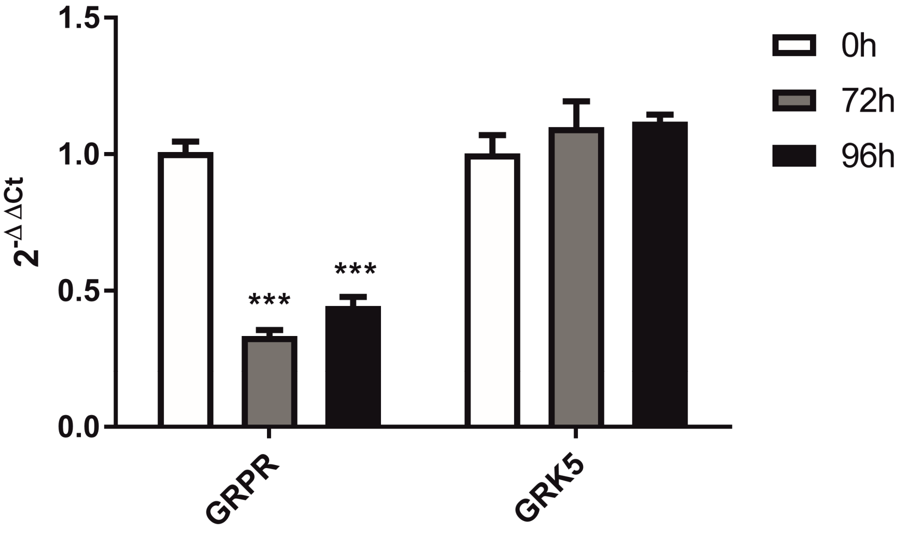

Supplementary figure S3. qPCR analysis of GRPR and GRK5. Examination of gene expression 72h and 96h after siGRPR transfection in MDA-MB-231 cells.
